# Function of the Airway Epithelium in Asthma

**DOI:** 10.1155/2012/160586

**Published:** 2012-03-22

**Authors:** Teal S. Hallstrand, Prescott G. Woodruff, Stephen T. Holgate, Darryl A. Knight

**Affiliations:** ^1^Division of Pulmonary and Critical Care Medicine, University of Washington, P.O. Box 356522, 1959 NE Pacific Street, Seattle, WA 98195-6522, USA; ^2^Division of Pulmonary and Critical Care Medicine, Department of Medicine, and Cardiovascular Research Institute, University of California, San Francisco, CA 94143, USA; ^3^Division of Anesthesiology, Pharmacology and Therapeutics, James Hogg iCAPTURE Centre for Cardiovascular and Pulmonary Research, University of British Columbia, Vancouver, BC, Canada V6Z 1Y6; ^4^Division of Infection, Inflammation and Immunity, University of Southampton School of Medicine, Southampton General Hospital, Southampton SO16 6YD, UK

An increasingly strong body of evidence implicates the airway epithelium as a critical regulator of airway inflammation and remodeling relevant to the pathogenesis of asthma. The evidence implicating the epithelium includes *in vivo* studies in humans and in murine models as well as *in vitro *studies conducted with primary epithelial cells. The purpose of this special issue is to provide a forum to integrate new knowledge about the nature of epithelial dysfunction in asthma and examine the role of the epithelium as a regulator of immune function. The articles contained within this special issue expound upon human studies examining epithelial gene expression, alterations in epithelial structure, as well as *in vitro* studies investigating epithelial function relevant to asthma. 

This special issue contains 3 original research articles and 4 review articles including some that delineate larger bodies of work by well-established research labs. The review articles focus on mechanisms related to the epithelium including the response to fungal allergens, epithelial apoptosis, production of mediators involved in remodeling of the matrix, and the important role of epithelial injury and the production of IL-25 in the induction phase of asthma. Two of the research articles highlight pathways of epithelial activation leading to inflammation that diverges from the traditional Th2 paradigm. In the final research article, the authors examine the utility of *in vitro* epithelial models.

In the paper entitled “*Mechanisms of remodeling in asthmatic airways,*” A. Shifren et al. from the group at Washington University including Dr. Mario Castro and others present an overview of structural changes that occur in patients with asthma and are particularly prominent in patients with severe asthma. In particular, their review outlines several mechanisms of epithelial activation leading to these structural changes in the subepithelial matrix as well as mucous cell alterations leading to further airflow obstruction. The roles of various therapies that may target remodeling are discussed.

In the paper “*Apoptosis and the airway epithelium*,” S. R. White from the University of Chicago presents a thorough overview of the function of epithelial apoptosis in health and disease states focusing on asthma, but also extending to other pulmonary diseases. The article has an outstanding overview of the function of apoptosis in the epithelium in contrast to immune cells and the basic mechanisms that regulate apoptosis in the epithelium. The article clearly delineates alterations in apoptosis that have been identified in human studies and *in vivo* animal models and the reasons that these alterations in the epithelium may play a pathogenic role in asthma and other lung diseases involving the epithelium.

In the paper “*Responses of airway epithelium to environmental injury: role in the induction phase of childhood asthma*,” R. K. Kumar and others from the University of New South Wales along with P. Foster and others from the University of Newcastle present a detailed picture of the factors involving the epithelium that may initiate airway inflammation. In particular, the response of the epithelium to viral infection and environmental pollutants such as diesel exhaust particles are presented as common triggers that occur in the epithelium leading to the generation of important epithelial factors including interleukin 25 (IL-25) and thymic stromal lymphopoietin (TSLP). The authors present evidence from their own work and others using studies in epithelial model systems as well as *in vivo* studies.

The final review article “*Immunopathology and immunogenetics of allergic bronchopulmonary aspergillosis*,” was written by A. P. Knutsen from the Department of Pediatrics at Saint Louis University. The review focuses on the pathogenesis of allergic bronchopulmonary aspergillosis (ABPA), a disease characterized by fungal hypersensitivity associated with central bronchiectasis and markedly elevated IgE. By relating a significant body of work relating to the epithelial response to aspergillus and other mold antigens, the author presents compelling evidence that the epithelium plays a key role in the pathogenesis of ABPA by regulating lymphocyte trafficking and activation.

In the original research article titled, “*Immunolocalization of NLRP3 inflammasome in normal murine airway epithelium and changes following induction of ovalbumin-induced airway inflammation*,” H. B. Tran et al. from the University of Adelaide use the murine model of ovalbumin-induced airway inflammation to examine the activation of the NLRP3 inflammasome in the airway epithelium. Little is known about the activation of this aspect of the innate immune system in asthma or model systems of asthma. In the epithelium, the authors found evidence of active caspase-1 and a redistribution of caspase-1, IL-1*β*, and IL 18 towards the luminal surface following sensitization and challenge with ovalbumin. These intriguing results should spur more research into triggers of asthma that may lead to inflammasome activation and the specific role of the inflammasome in models of asthma.

In the research paper “*IL-17F induces CCL20 in bronchial epithelial cells*,” K. Nozato et al. examine the regulation of CCL20 expression by IL-17F in bronchial epithelial cells. The authors demonstrate that IL-17F modulates CCL20 expression through a mitogen-activated protein kinase (MAPK) pathway leading to the activation of the cyclic AMP response element-binding (CREB) transcription factor. This paper adds to other evidence by this investigative team demonstrating that IL-17F induces CXC chemokines, GM-CSF, IP-10, IL-11, and IGF-1 through this pathway.

In the final research paper “*Evaluation of differentiated human bronchial epithelial cell culture systems for asthma research*,” C. F. Stewart et al. from the University of Nottingham working with I. Sayers evaluated differences between *in vitro* models of the epithelium. The investigators used primary human bronchial epithelial cells as well as several cell lines including a lung adenocarcinoma cells line (Calu-3) and SV-40-transformed human bronchial epithelial cells (BEAS-2B). Using these cells they found that there were differences in markers of differentiation such as mucous cell differentiation between the cultured cells, as well as differences between the junction proteins. Although the basis of the differences are is know in detail, the research found that there were differences between primary epithelial cells donor as well as between cultures that originated from the same donor raising important questions about the variability of these model systems.

Taken together, these articles provide yet further evidence that in asthma the epithelium takes center stage in orchestrating responses to the inhaled environment through pathways influencing inflammation as well as aberrant repair. In addition, the epithelium provides a platform for identifying novel therapeutic targets linked to increasing the resistance of the airways to environmental injury. We are exceedingly grateful to the many leaders in the field of epithelial biology and asthma who have contributed to this special issue. The strong response to this call for papers by well-recognized researchers in this field provides additional evidence of the emerging importance of the field of epithelial biology to the understanding of asthma.

*Teal S. Hallstrand* *Teal S. Hallstrand* *Prescott G. Woodruff* *Prescott G. Woodruff* *Stephen T. Holgate* *Stephen T. Holgate* *Darryl A. Knight* *Darryl A. Knight*

